# Investigating the Mechanisms Underlying Citral-Induced Oxidative Stress and Its Contribution to Antifungal Efficacy on *Magnaporthe oryzae* Through a Multi-Omics Approach

**DOI:** 10.3390/plants14132001

**Published:** 2025-06-30

**Authors:** Yonghui Huang, Ruoruo Wang, Yumei Tan, Yongxiang Liu, Xiyi Ren, Congtao Guo, Rongyu Li, Ming Li

**Affiliations:** 1 Guizhou Institute of Biotechnology, Guizhou Academy of Agricultural Sciences, Guiyang 550006, China; 2 Key Laboratory of Crop Genetic Resources and Germplasm Innovation in Karst Region, Ministry of Agriculture and Rural Affairs, Guiyang 550006, China; 3 Guizhou Key Laboratory of Agricultural Microbiology, Guiyang 550006, China; 4 Institute of Crop Protection, College of Agriculture, Guizhou University, Guiyang 550025, China

**Keywords:** citral, *Magnaporthe oryzae*, antifungal activity, oxidative stress, widely-targeted metabolome, transcriptome

## Abstract

Citral, an organic compound found in lemongrass (*Cymbopogon citratus*) oil and *Litsea cubeba* essential oil, has been reported to exhibit notable antifungal activity against *Magnaporthe oryzae* (*M. oryzae*), the pathogen of rice blast, which causes significant economic losses in rice production. However, the role of citral in inducing oxidative stress related to antifungal ability and its underlying regulatory networks in *M. oryzae* remain unclear. In this study, we investigated the oxidative effects of citral on *M. oryzae* and conducted transcriptomic and widely targeted metabolomic (WTM) analyses on the mycelia. The results showed that citral induced superoxide dismutase (SOD), catalase (CAT), ascorbate peroxidase (APX) activities but reduced glutathione S-transferase (GST) activity with 25% maximal effective concentration (EC_25_) and 75% maximal effective concentration (EC_75_). Importantly, citral at EC_75_ reduced the activities of mitochondrial respiratory chain complex I, complex III and ATP content, while increasing the activity of mitochondrial respiratory chain complex II. In addition, citral triggered a burst of reactive oxygen species (ROS) and a loss of mitochondrial membrane potential (MMP) through the observation of fluorescence. Furthermore, RNA-seq analysis and metabolomics analysis identified a total of 466 differentially expression genes (DEGs) and 32 differential metabolites (DAMs) after the mycelia were treated with citral. The following multi-omics analysis revealed that the metabolic pathways centered on AsA, GSH and melatonin were obviously suppressed by citral, indicating a disrupted redox equilibrium in the cell. These findings provide further evidences supporting the antifungal activity of citral and offer new insights into the response of *M. oryzae* under oxidative stress induced by citral.

## 1. Introduction

Rice blast caused by *Magnaporthe oryzae* is the most common and deadly disease affecting rice yield and quality in the world, which results in significant damage to rice yield reduction (40~50%) or even no harvest in severe cases [1]. The infection of *M. oryzae* occurs through the whole growth cycle of rice, and spreads and reproduces through hyphae and conidia, making rice blast management very difficult [2,3,4]. As one of the main rice planting countries, China has also suffered from rice blast disease all year round, with an annual production reduction of millions or even tens of millions of tons. At present, the primary efficient way for controlling blast disease is cultivating disease-resistant varieties supplemented by precise chemical control [5,6]. However, due to the reckless application of chemical fungicides throughout years, we have surfaced a series of risks such as the toxicity to non-targeted species, fungicide’s resilient development and environment pollution [7,8]. Under this circumstance, it is important to seek biodegradable and environmentally friendly alternatives; a number of promising natural candidates for combating microbial infections have emerged.

Essential oils derived from plants consist of lipophilic and highly volatile secondary metabolites such as active aldehydes, terpenes, ketones, alkaloids, flavonoids, steroids, phenols and acids [9,10]. Currently, due to the complex issues brought about by massive chemical preservation and pesticide applications, essential oils and their isolates have aroused great interest in the development of medicine, food preservation and pesticides. They exhibit unique biological activities like antioxidant, insecticidal, antifungal and antibacterial functions [11,12,13,14]. These bioactive compounds are considered to have muti-targeting activity and low host cytotoxicity, making them a good resource for low-toxicity pesticides and synergists [15,16].

Citral, the main active component of lemongrass (*Cymbopogon citratus*) essential oil and *Litsea cubeba* essential oil, has been reported to have an excellent inhibitory effect against various bacteria and fungi [9,17,18,19]. Actually, citral (3,7-dimethyl-2,6-octadienal) is a natural mixture of two isomeric acyclic monoterpene aldehydes, geranial (trans-citral, citral A) and neral (cis-citral, citral B). As an unsaturated monoterpene, citral is capable of trapping activated oxygen species in vivo to give intermediate epoxides, which can alkylate DNAs, proteins, and other biomolecules [20]. Studies have demonstrated that citral can damage the fungal cell wall and the cell membrane of *Candida albicans*, *Magnapothe oryzae*, *Penicillium roqueforti*, *Aspergillus flavus* and *Penicillium digitatum*, *Aspergillus niger* ect., which results in morphological change, leakage of cellular macromolecules and cell death [21,22,23,24,25]. In addition, citral can not only trigger oxidative stress, which cause mitochondrial dysfunction, but also activate cell cycle arrest and apoptosis-signaling pathways in fungi [26,27,28,29,30]. However, in the antifungal research of citral, previous studies have mainly focused on revealing its activity against various fungi, as well as related morphological and physiological changes, yet the changes in fungal genes and metabolites and gene–metabolite crosstalk in response to oxidative stress caused by citral have rarely been studied. This study aimed to investigate the response of *M. oryzae* to oxidative stress induced by citral and gain deeper insight into its molecular mechanisms. Our previous work has shown that citral acts by disrupting cell wall integrity and membrane permeability, resulting in physiology changes and causing cytotoxicity [21,31]. We speculated that citral may trigger ROS in *M. oryzae* cells and destroy the redox equilibrium, which is responsible for induction of cellular apoptosis. In the experiment design, 25% maximal effective concentration (EC_25_) and 75% maximal effective concentration (EC_75_) were chosen to evaluate sub-lethal and high-effect thresholds in consideration of their well-established practices in toxicology and pharmacology studies [32,33]. By treating *M. oryzae* mycelia with citral at EC_25_ and EC_75_ for a series of hours, we investigated antioxidant enzymatic activities, reactive oxygen species (ROS) burst, mitochondrial membrane potential (MMP) and mitochondrial respiration chain complexes activities. To delve into the underlying mechanism, we conducted comparative transcriptomic and widely targeted metabolomic (WTM) analysis on citral-treated mycelia and controls. The findings of our present study could provide better elucidation of the antifungal mechanism of citral and hold great potential for developing plant derived pesticides.

## 2. Results

### 2.1. Citral Affected the Activities of Antioxidant Enzymes in M. oryzae

**Figure 1:**
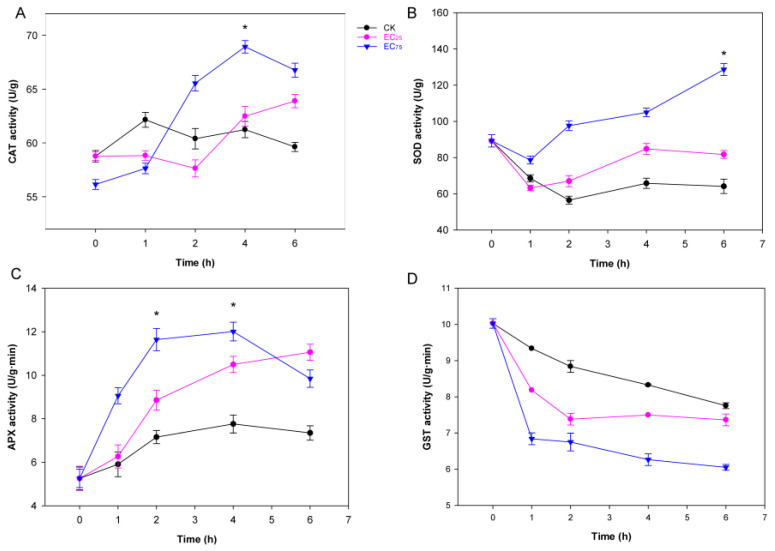
Effects of citral on activities of antioxidant enzymes in *M. oryzae*. (**A**) Change in CAT activity; (**B**) change in SOD activity; (**C**) change in APX activity; (**D**) change in GST activity. Cells of *M. oryzae* ZB08 were treated with citral at EC_25_ (19.83 µg/mL) and EC_75_ (110.95 µg/mL), whereas untreated cells served as control group. Error bars represent the SD, and each point is the average of triplicate cultures. Asterisks indicate a statistically significant difference (*p* < 0.05).

Antioxidant enzymes can effectively inhibit the oxidative process in organisms and prevent the harm caused by peroxidation. The activities of catalase (CAT), superoxide dismutase (SOD), ascorbate peroxidase (APX) and glutathione-S-transferase (GST) were tested to briefly check the fungal defense in *M. oryzae*. As shown in Figure 1, the activities of CAT and SOD in *M. oryzae* exposed to citral generally increased along with treatment time. Under the treatment of citral at EC_75_, the activities of CAT in *M. oryzae* quickly achieved a peak (68.93 ± 0.58 U/g) at 4 h, and decreased in the remaining 2 h, while under the treatment of citral at EC_25_, CAT activity of *M. oryzae* rose for 6 h except for a transient decline at 2 h. The SOD activity of *M. oryzae* exposed to citral at EC_75_ increased consistently in 6 h treatment with a dramatic change, and a relatively smaller variation was shown in that of EC_25_. When exposed to citral at EC_75_, the APX activity of *M. oryzae* exponentially rose in the first 2 h and reached a peak of 12.01 ± 0.43 U/g in 4 h treatment, ending with a decline at 6 h. In EC_25_ treatment, the APX activity constantly increased and finally reached 11.06 ± 0.37 U/g at 6 h. However, the GST activities of mycelia in the control group and citral-treated groups decreased in the first hour, but the two citral-treated groups dropped sharply. Compared with the control group, the GST activity of mycelia treated with EC_25_ and EC_75_ decreased by 12.3% and 23.3%, respectively. In the final stage, the downward trend of the two treatment groups decelerated, and the GST activity of EC_25_ group showed a slight increase. Nevertheless, the GST activities of the control group remained higher than that of the treatment groups throughout the entire treatment period. The results indicated that citral could significantly induce the activities of CAT, SOD and APX, but reduce the activity of GST in *M. oryzae*.

### 2.2. Citral Affected Mitochondrial Function and ATP Synthesis in M. oryzae

**Figure 2:**
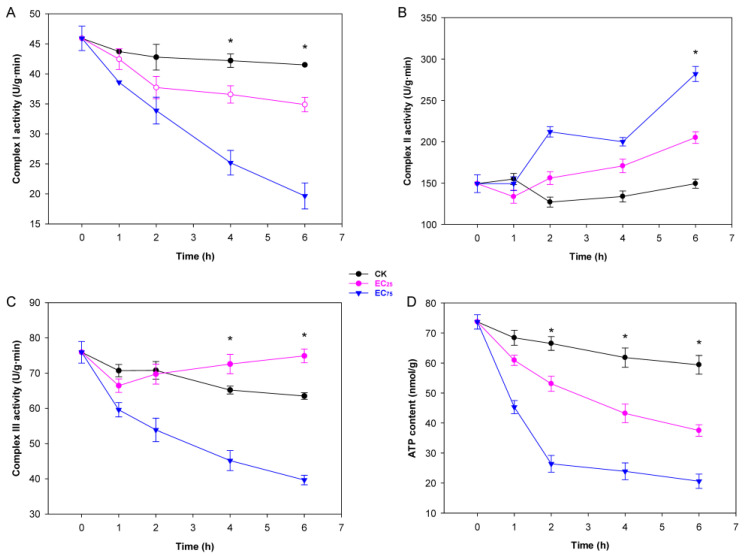
Effects of citral on activity of mitochondrial respiration chain complex I, complex II, complex III and ATP content in *M. oryzae*. (**A**) Change in mitochondrial respiration chain complex I activity; (**B**) change in mitochondrial respiration chain complex II activity; (**C**) change in mitochondrial resparation chain complex III; (**D**) change in ATP content. Cells of *M. oryzae* ZB08 were treated with citral at EC_25_ (19.83 µg/mL) and EC_75_ (110.95 µg/mL), whereas untreated cells served as control group. Error bars represent the SD and each point is the average of triplicate cultures. Asterisks indicate a statistically significant difference (*p* < 0.05).

Mitochondrial respiration chain complex (Mt complex) I, complex II, and complex III in the inner membrane of mitochondria, working as a proton pump, are vital for energy storage during oxidative phosphorylation and ATP synthesis [34]. After treatment with citral, the activity of Mt complex I was significantly inhibited, especially in EC_75_, which linearly decreased throughout the exposure time, ending with a lowest value of 19.66 ± 2.16 U/g (Figure 2A). In contrary to complex I, Mt complex II activity was increased by citral and reached peaks of 199.98 ± 5.11 U/g and 282.0 ± 9.15 U/g at EC_25_ and EC_75_, respectively, after 6 h of treatment (Figure 2B). The activity of Mt complex III was significantly inhibited when treated with citral at EC_75_, while that of EC_25_ treatment seemed slightly induced (Figure 2C). Besides the effect on Mt complexes, both low concentration (EC_25_) and high concentration (EC_75_) of citral treatment caused significant inhibition of the intracellular ATP content. Compared to control mycelia of *M. oryzae*, the reduction rates in EC_25_ and EC_75_ of citral were 65% and 36.9%, respectively, after 6 h of treatment (Figure 2D). Together, the results suggest that citral-induced mitochondrial dysfunction directly depletes intracellular ATP pools, crippling energy-dependent processes essential for the survival of *M. oryzae*.

### 2.3. Citral Affected ROS Accumulation in M. oryzae

**Figure 3:**
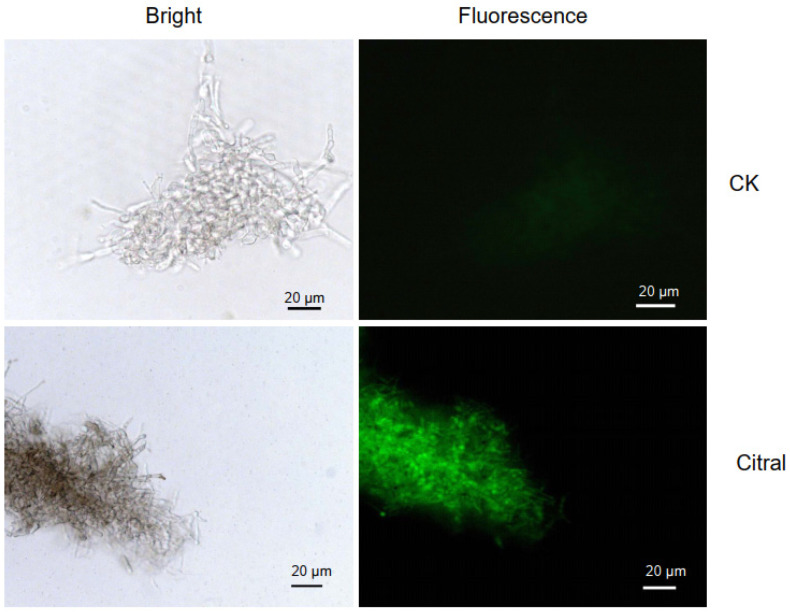
Effects of citral on ROS accumulation of *M. oryzae* detected by fluorescence microscopy using DCFH-DA. Mycelia of *M. oryzae* ZB08 were treated with citral at EC_75_ (110.95 µg/mL) for 4 h whereas cells treated with equal solvent served as control group. Images were observed using fluorescence microscope.

Reactive oxygen species (ROS) are a natural byproduct of routine oxygen metabolism and play an important role in cellular signaling and homeostasis. ROS levels can sharply increase during periods of environmental stress and cause serious damage to cellular structure and function, and high accumulation of ROS upon oxidative stress can lead to damage of cell membranes and biomolecules, consequently leading to cell apoptosis [35]. Here, the change in ROS accumulation in *M. oryzae* was visually detected with dichloro-dihydro-florescein diacetate (DCFH-DA), which can freely penetrate into cells and then be hydrolyzed into DCFH by intracellular esterases. DCFH cannot penetrate the cell membrane, and so can be oxidized by ROS within cells to generate fluorescent DCF. During the experiment, after treatment with citral for 1 h and 2 h, slightly stronger fluorescence was observed in the mycelia compared to the control. Much greater change occurred after treatment with citral for 4 h; the untreated mycelia emitted weak fluorescence, while the citral-treated mycelia produced obviously enhanced fluorescence (Figure 3). This observation demonstrated that citral trigged ROS burst in the mycelia of *M. oryzae*, indicating that citral induced excessive oxidative stress and disrupted redox balance in the cells of *M. oryzae*.

### 2.4. Citral Affected the MMP of M. oryzae

**Figure 4:**
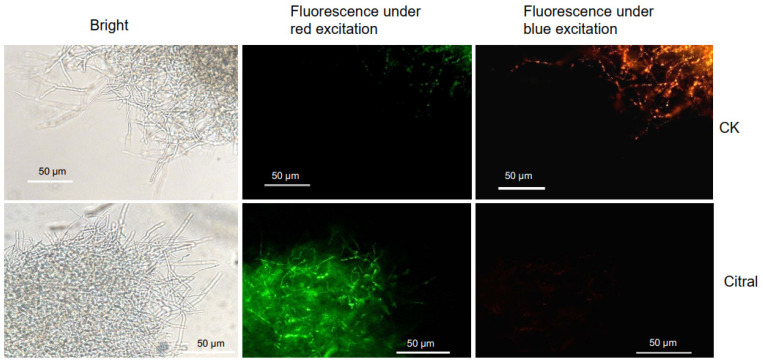
Effects of citral on MMP of *M. oryzae* detected by fluorescence microscope using JC-1. Mycelia of *M. oryzae* ZB08 were treated with citral at EC_75_ (110.95 µg/mL) for 4 h, whereas mycelia treated with equal solvent served as control group. Images observed using fluorescence microscope.

Mitochondrial membrane potential (MMP), which might be caused by the stimulus of ROS, is a key indicator of cell health and mitochondrial function. Before the appearance of apoptotic features such as chromatin condensation and DNA fragmentation, the collapse of MMP marks the irreversibility of cell apoptosis [36]. To further investigate the physiological change in *M. oryzae* cells by citral, the MMP was examined by JC-1 staining. When MMP is high, JC-1 aggregates in the mitochondrial matrix, forming a polymer that can produce red fluorescence. When the MMP is low, JC-1 cannot aggregate in the mitochondrial matrix but exists as a monomer and produce green fluorescence. In the early 1-2 h of incubation with citral at EC_75_, red fluorescence was easily observed, while green fluorescence was difficult to be detected. However, significant changes occurred after the mycelia were treated with citral for 4 h. In the control group, *M. oryzae* mycelia displayed relatively stronger spontaneous red fluorescence than green fluorescence, indicating that the MMP was high. *M. oryzae* cells displayed significantly stronger green fluorescence and weaker red fluorescence compared to control mycelia, which meant a significant depolarization in MMP (Figure 4). The results indicated that citral caused a decrease in MMP, which prefigured a breakdown in mitochondrial energy metabolism, the activation of apoptotic signals and exacerbation of pathological damage.

### 2.5. The Response of Transcriptome in M. oryzae to Citral Exposure

#### 2.5.1. Illumina Sequencing and Sequence Assembly

To investigate the molecular mechanisms of how *M. oryzae* respond to citral, transcriptome analysis was performed on the mycelia. After incubation with citral at EC_75_ for 4 h, RNA was extracted with samples of citral-treated and control mycelia. All qualified total RNA samples were applied to construct a total of 6 RNA-seq libraries and sequenced to identify the DEGs that were responsive to citral stress. After filtering the raw reads from sequencing, clean reads were obtained, with an average output of 6.75 Gb of data per sample (Table S1). The quality of filtered reads is shown in Table S1, with a clean reads Q20 average value of 98.37% and a clean reads Q30 average value of 95.27%. The average mapping rate of each sample reached 95.98%, and the mapping rate between samples was uniform, indicating that the data between samples was comparable. This suggests that the sequence quality was available for further bioinformatic analysis.

#### 2.5.2. Analysis of Differentially Expressed Genes

Compared to the control group, 118 genes were significantly up-regulated, and 348 genes were significantly down-regulated in the citral-treated mycelia. Volcanic diagram statistics and a heatmap of hierarchical clustering were used to clarify the overall status of DEGs (Figures S1 and S2). As shown in Figure S1, the differential expression levels of genes was mainly concentrated between 0.125 (log2 fold change = −3) and 8 (log2 fold change = 3) times, and the differences were significant (*p* value < 0.05). The relative gene expression levels of selected genes were determined by quantitative real-time polymerase chain reaction (qRT-PCR), and the results were consistent with RNA-seq (Table S2, Figure S3).

#### 2.5.3. GO Functional Analysis and KEGG Enrichment Analysis

**Figure 5:**
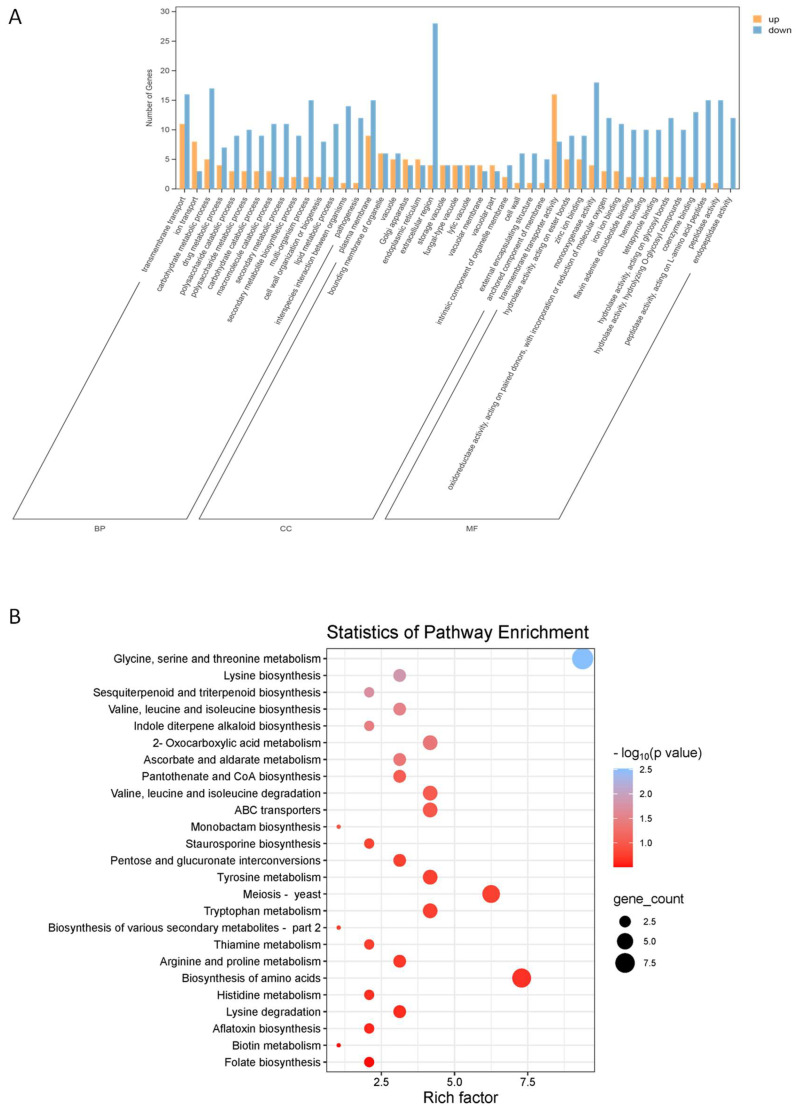
Transcriptomic analysis between citral-treated mycelia and control mycelia of *M. oryzae*. (**A**) GO functional enrichment of DEGs in *M. oryzae*. BP, biological process; CC, cell component; MF, molecular function; (**B**) KEGG pathway enrichment of DEGs in *M. oryzae*.

The overall GO classification was applied to analyze the functional distribution of DEGs. The results showed that DEGs were mostly enriched in biological processes (BPs), with metabolic processes and cellular process as the dominant two groups. The second category was cellular component (CC), and substantial DEGs were mainly related to the cell/cell part and cell membrane/cell membrane part. The third category was molecular function (MF), with an outstanding DEG aggregation in catalytic activity and binding (Figure 5A). The top 50 significantly concentrated GO functional terms showed that there were 141 DEGs enriched in BP, and the main terms were transmembrane transport, carbohydrate metabolic process, multiorganism process and interactions between organisms (Figure S4). One hundred and seventy-nine DEGs were enriched in CC, and the main enriched terms were related to the cell wall and plasma membrane. MF enriched a total of 191 DEGs, mainly including various transmembrane transporter activity, monooxygenase activity, peptidase activity, oxidoreductase activity and metal ion binding.

Furthermore, the biochemical metabolic pathways and signal transduction pathways involved in DEGs were analyzed using the KEGG database to evaluate their response to citral. The results showed that the gene expression of *M. oryzae* cells was disrupted after 4 h exposure to citral, leading to the global regulation of metabolic processes. A total of 96 DEGs were identified in 77 different KEGG pathways, of which 69 DEGs were involved in the metabolic pathway, and 28 DEGs were involved in the biosynthesis pathway of secondary metabolites. Except for these two putative pathways with high rich factor, the other significantly enriched 20 pathways are shown in Figure 5B. Among them, glycine, serine, and threonine metabolism, lysine biosynthesis, sesquiterpenoid and triterpenoid biosynthesis, valine, leucine, and isoleucine biosynthesis, indole diterpene alkaloid biosynthesis, 2-oxocarboxylic acid metabolism, and ascorbate and aldarate metabolism were the top enriched pathways. The above 7 pathways could be summarized as amino acid biosynthesis, oxidation-reduction, biosynthesis of terpenoids and their derivatives. The results suggested that citral induced oxidative stress to *M. oryzae*, as reflected in pathways involved in redox equilibrium, amino acid metabolism and other pathways that may respond to external stress.

### 2.6. The Response of Metabolome in M. oryzae to Citral Exposure

#### 2.6.1. Metabolite Detection and Clustering Analysis of Differentially Accumulated Metabolites (DAMs)

Based on widely targeted metabolomics technology, a total of 435 metabolites were detected in metabolic analysis with UPLC-MS/MS platform. The DAMs modulated by citral exposure were investigated using a self-building database of Wuhan Metware Biotechnology Co., Ltd. (Wuhan, China) and multivariate statistical analysis. Principal component analysis (PCA) was performed, and the score plot (Figure S5a) showed that the raw data obtained from UPLC-MS/MS analysis was well presented in both PC1 and PC2 principal components. The two groups of samples showed a clear separation trend on the two-dimensional graph, indicating that the data-processing results for each sample were reliable, and there were significant differences between the sample groups. The OPLS-DA model was subjected to 200 rounds of permutation validation, and the results showed that it had good predictive ability and reliability, and thus could well represent the trend of metabolite changes between groups (Figure S5b). The results showed that there were 32 significant DAMs after treatment with citral at EC_75_ for 4 h, including 11 up-regulated metabolites and 21 down-regulated metabolites. These DAMs contained nine organic acids, five benzenes and its derivatives, four amino acids, three nucleotides, three coenzymes and vitamins, one glycerol, two fatty acids, two hormones and two heterocyclic substances. The most down-regulated metabolite of *M. oryzae* was melatonin, with a Log2FC of −11.29, while the most up-regulated was 4-methylbenzoic acid, with a Log2FC of 12.79 (Table 1). These significant DAMs related to biological metabolisms were clustered together and visualized through a heatmap of hierarchical clustering (Figure S6).

#### 2.6.2. KEGG Enrichment Analysis of DAMs

**Figure 6:**
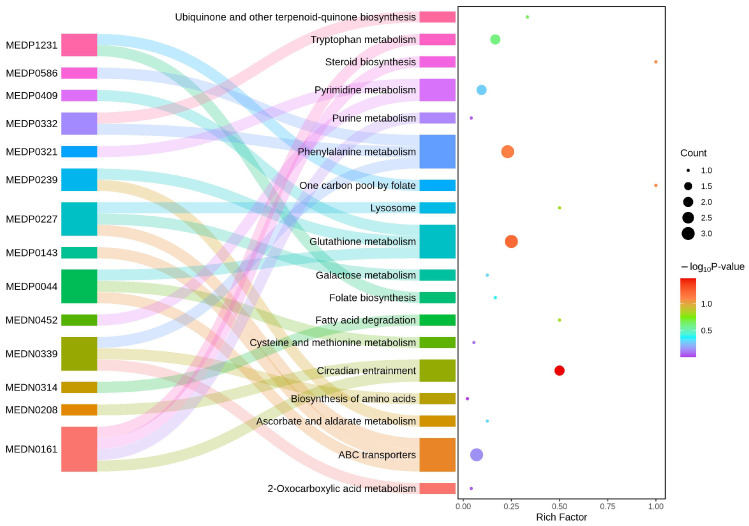
Sanky plot for KEGG pathway enrichment analysis of DAMs between citral-treated and control groups of *M*. *oryzae* mycelia. Pathways for plot were selected by the enrichment level, and the top 18 ones with their related metabolites are shown.

The further KEGG pathway enrichment analysis presents a Sankey plot showing the enriched pathways and their associations with DAMs (Figure 6). The results showed that the DAMs significantly enriched in circadian entrainment pathway, glutathione metabolism pathway and phenylalanion metabolism pathway, which had the largest red bubbles. Among the significant DAMs, compound MEDN0161 (melatonin) was noticed for its flux to several pathways including circadian entrainment, purine metabolism, tryptophan metabolism and steroid biosynthesis; compound MEDP0044 (glutathione reduced form) was related to glutathione metabolism, cystein and methionine metabolism and ABC transporters; compound MEDP0239 (L-ascorbate) was involved in ascorbate and aldarate metabolism and glutathione metabolism. Considering their functions in redox balance as reported, we inferred that melatonin, glutathione and ascorbate may play a crucial role in the response of *M*. *oryzae* to oxidative stress caused by citral. We also noticed that some insignificant DAMs, such as MEDP0227 (D-glucose 6-phosphate) and MEDN0339 (phenylpyruvic acid), were also involved in the enriched pathways like phenylalanine metabolism and the biosynthesis of amino acid and ABC transporters, as they play important roles in biological processes.

### 2.7. Association Analysis of Transcriptome and WT Metabolome

Based on the enrichment analysis results for the DEGs and DAMs, we found that they shared 15 enriched KEGG pathways, which was shown in a bar chart drawn to display their association (Figure S5). The relatively enriched pathways were concentrated on glutathione metabolism, phelylalanine metabolism, glycine, serine and threoine metabolism, 2-oxocarboxylic acid metabolism pathways in genes and metabolites. The results suggested that citral mainly affected the metabolism of amino acids and redox equilibrium in *M*. *oryzae*.

**Figure 7:**
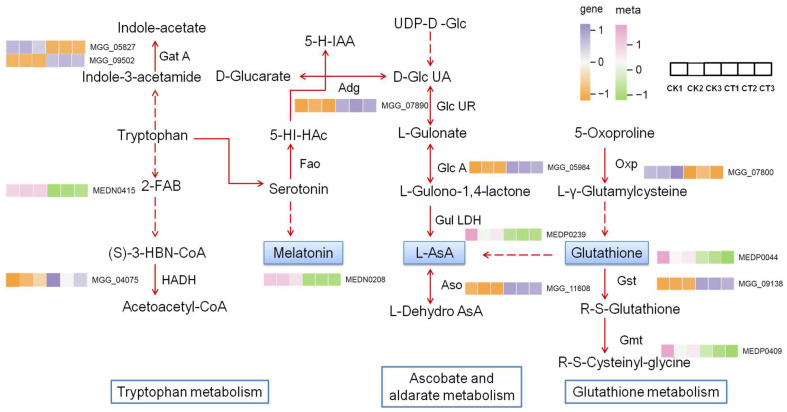
An illustrative scheme of gene expression and metabolite changes in the KEGG pathways of tryptophan metabolism, ascorbate and aldarate metabolism and glutathione metabolism between mycelia treated with citral at EC_75_ and control mycelia in *M. oryzae*. CK, control group; CT, citral-treated group; Gat A, glutamyl-tRNA (Gln) amidotransferase subunit A; HADA, 3-hydroxyacyl-CoA dehydrogenase; Adg, aldehyde dehydrogenase; Fao, flavin-cotaining amine oxidase; Glc UR, glucuronic reductase (NADP-dependent alcohol dehydrogenase); Glc A, gluconolactonase lactonase; Gul LDH, L-gulonolactone oxidase; Aso, laccase; Oxp, oxoprolinase; Gst, glutathione S-transferase; Gmt, glutathione hydrolase. The up-regulated and down-regulated genes and metabolites are shown via heatmap. The dotted arrows indicate the biosynthesis of the metabolite is a multipath.

To further understand the interaction between DEGs and DAMs, an integrated oxidation–reduction system centered on three key metabolites, melatonin, L-ascorbate and glutathione, as well as the related pathways was constructed, and this is shown in an illustrative scheme (Figure 7). After treatment with citral, the three key metabolites were down-regulated in the system, of which melatonin was the top significantly down-regulated one (Table 1). Besides them, 2-(formylamino) benzoic acid (MEDN0415) in the tryptophan metabolism pathway and cysteinyl-glycine (MEDP0409) in the glutathione metabolism pathway were down-regulated too. The expression levels of 3-hydroxyacyl-CoA dehydrogenase (MGG_04075), glutamyl-tRNA(Gln) amidotrans-ferase (MGG_09502), aldehyde dehydro-genase (MGG_07890), gluconolactonase lactonase (MGG_05984), laccase (MGG_11608) and glutathione S-transferase (MGG_09138) were up-regulated, while that of glutamyl-tRNA(Gln) amidotransferase subunit A (MGG_05827) and oxoprolinase (MGG_07800) were down-regulated. In the illustrated redox system, all the DAMs were down-regulated, while the expression levels of most genes show an up-regulated trend. Up-regulation of aldehyde dehydrogenase might lead to a decrease in serotonin and so reduce the synthesis of melatonin, whereas the up-regulation of glutathione S-transferase and down-regulation of oxoprolinase might indirectly depress the accumulation of glutathione. Interestingly, the gene expression pattern of GST and GSH content are contrary to enzyme activity in our study. The synchronous decrease in GST activity and GSH content might be considered the comprehensive manifestation of the redox imbalance and the breakdown of detoxification function in citral-treated cells of *M*. *oryzae*, but the enhancement in gene expression level may be a compensation for the decrease in enzyme activity.

## 3. Discussion

Oxidative stress occurs due to an imbalance between reactive oxygen species (ROS) and antioxidants. Under abiotic and biotic stress conditions, high levels of ROS generation cause an oxidative stress situation that includes damaging cellular components like lipids, proteins, and DNA. Fungi exhibit complex mechanisms to balance ROS production and detoxification, which is crucial for their survival, pathogenicity, and ecological roles. Enzymes like SOD, CAT and GST are employed by fungi to scavenge superfluous ROS, thus protecting cell and subcellular systems [37,38]. Our results in the antioxidant enzymatic assay showed that activities of SOD, CAT and APX were significantly enhanced when treated with citral at EC_75_ concentration, whereas GST activities were depressed in this case (Figure 1). The activation of primary ROS-detoxifying enzymes such as CAT and SOD by varied means has been evidenced in fungal tolerance to oxidative stress [16,39,40]. Consistent with the activity performance, the up-regulation of *sod* gene (MGG_13253) was also found in the transcriptomic data of citral-treated mycelia, but there was no change in the expression level of *cat* and *apx* genes. Although the enzyme activities of GST decreased after treatment with citral, the *gst* gene (MGG_09138) was up-regulated at the transcriptomic level, indicating a compensation for GST synthesis in *M*. *oryzae*. Combined with the ROS fluorescence observation (Figure 3), the hypothesis that citral induces oxidative stress to *M*. *oryzae* is validated.

Moreover, oxidative stress significantly impacts fungal mitochondria through affecting their structure, function, and overall cellular health. Firstly, ROS attacks unsaturated fatty acids in the mitochondrial inner membrane, causing damage to membrane integrity and membrane potential, leading to ATP synthesis failure. Secondly, ROS oxidize iron-sulfur clusters in electron transport chain complexes (Mt I, II, III), disrupting electron flow and exacerbating ROS leakage [16,27]. Our results showed that ROS caused by citral could reduce the activities of Mt complex I and Mt complex III and thus inhibit ATP synthesis, although the activities of Mt complex II exhibited a contrary enhancement (Figure 2). The decrease in ATP content may also be related to the damage of MMP in *M*. *oryzae*, which is caused by the damage of citral to mitochondrial membrane (Figure 4). In our early study, we found that citral caused the breakage of cell membranes of *M. oryzae* through morphological observation and detection of an increase in soluble protein, reducing sugar and MDA [22]. It can be summarized that oxidative stress disrupts the integrity of cell and mitochondrial membranes in *M. oryzae* and changes in membrane permeability, resulting in energy metabolism breakdown and thus limiting fugal growth. It is noteworthy that although there was a notable alteration in activities of Mt complexes within the mycelia treated with citral, the gene expression levels of the three complexes remained unchanged in RNA-seq analysis, being absent from the DEG list. This phenomenon may arise because redox responses can occur through enhanced mRNA translation efficiency rather than transcriptional upregulation. Additionally, ROS-generating enzymes may be activated via existing protein modification, such as phosphorylation or oxidation, without requiring de novo gene expression.

The comparative RNA-seq analysis of citral-treated mycelia and control mycelia of *M. oryzae* displayed the oxidative stress causing changes in gene expression level. The KEGG enrichment analysis showed that pathways associated with amino acids synthesis or metabolism, such as glycine, serine and threonine metabolism, lysine biosynthesis, valine, leucine, and isoleucine biosynthesis, were prominent changes in response to citral-triggered oxidative stress (Figure 5B). Amino acids are integral to eukaryotic strategies against oxidative stress, and its biosynthesis could be activated in order to serve as antioxidants, enzyme components, and metabolic regulators [41,42]. The three amino acids involved in the metabolism of glycine, serine, and threonine are crucial for organisms to cope with stress environments, as they participate in biochemical reactions such as glycine synthesis in GSH and regulate physiological processes such as supporting DNA repair by the release of one carbon unit [43,44]. In the current metabolic profile, Cys-Gly and GSH in *M. oryzae* were down-regulated after exposure to a high concentration of citral. This is due to excessive consumption of GSH caused by a surge in antioxidant demand, coupled with limited synthesis, leading to severe imbalance in the cellular metabolic network. Conversly, N-acetylthreonine was up-regulated, potentially reflecting a fungal compensatory mechanism to restore the GSH accumulation, given that threonine serves as a precursor for glycine (Table 1). Moreover, metabolomic profiling further revealed the up-regulation of N-acetylphelylalanine. Notably, phenylalanine (PA) functions as a potential amino acid scavenger of ROS, thereby mitigating oxidative damage [45,46]. The metabolomic and transcriptomic analysis of amino acids indicates that amino acids play an important role in the comprehensive response of *M. oryzae* to citral oxidative stress.

Molecules such as GSH, ASA, and thioredoxin can scavenge ROS, and the ascorbic acid–glutathione (GSH-ASA) cycle is an important antioxidant strategy for organisms to resist external stress. In our present results, the down-regulated ascorbate and alderate metabolism is among the most enriched KEGG pathways. As seen in Figure 6, not only did GSH and cys-gly decrease after exposure to citral, but also ASA was reduced. The GSH-ASA cycle is well established as a critical mechanism in preventing oxidative stress and preserving redox homeostasis. Consequently, reduced levels of GSH and ASA may trigger substantial accumulation of cellular oxidative damage. Vipul Mishra et al. found that abiotic stress in plants caused elevated reactive oxygen species levels and inhibition of ASA-GSH cycle enzyme activities as well as their relative gene expression, thus causing lipid and protein oxidation [47]. Similarly, when studying the toxicity of hexavalent chromium on two pulse crops, the levels of ASA and GSH may be one of the reasons for the higher accumulation of H_2_O_2_ [48]. Studies have shown that the way cells respond to ROS-induced oxidative stress may depend on the concentration or intensity of external stimuli. For example, low concentrations of CuCeOx exhibited proactive regulation of CAT and GSH levels to mitigate the deleterious effects of oxidative damage [49], and pre-treatment with triacontanol increased the AsA and GSH content and antioxidant enzyme activity in *Brassica napus* L., thereby reducing oxidative stress [50]. A more comprehensive study has demonstrated that under Cu toxicity in two cyanobacteria, UV-B radiation at low fluence rate stimulated protective responses while UV-B irradiation at high fluence rate caused damage to activities of antioxidant enzymes and components of AsA-GSH cycle [51]. Our results supported that citral at high concentrations (EC_75_) could cause the decline of the AsA-GSH cycle in *M. oryzae*, resulting in severe disruption of the redox balance.

In addition to AsA-GSH, more studies have revealed that melatonin (Mel) also plays a regulatory role in redox equilibrium. It is a bioactive substance with multiple physiological functions, including regulating circadian rhythms, enhancing immune function, strengthening antioxidant function, and regulating reproductive system function. Supplementing 30 µM Mel to drought-stressed plants improved the activities of ATP synthase and ATPase, activated the ASA-GSH system and antioxidant enzymes, and increased the accumulation of osmolytes [52]. Mel plays an important regulatory role in fungi under abiotic stress. Studies have shown that under low temperature and low sugar conditions, the peak level of intracellular Mel in brewing yeast cells was delayed, while the synthesis of Mel significantly increased at a moderate temperature of 12 °C [53,54]. In *Tolypocladium guangdongense*, low-temperature stress, high-temperature stress, and Congo red stress could significantly promote the production of intracellular Mel, while H_2_O_2_ stress tended to inhibit its synthesis [55]. Similar results were discovered with the increase in Mel levels in shiitake mushrooms under cadmium stress and low-temperature storage [56]. The mechanism of antioxidant function of Mel was not clear, but the increase in antioxidant enzymes and decrease in ROS accumulation might be one of the reasons [57]. Reduced GSH and increased ROS may exacerbate circadian rhythm abnormalities by inhibiting the expression of circadian rhythm genes [58,59]. In our study, citral induced a significant decrease in Mel levels in *M. oryzae*, indicating its critical role in the fungal response to oxidative stress. This decline in Mel paralleled the reduction trend of GSH and ASA, suggesting its contribution to the aggravation of redox imbalance under elevated citral concentration.

Since the discovery of citral’s inhibitory effect against *M. oryzae*, we have dedicated extensive research to elucidate its underlying mechanisms. In summary, citral breaks down the cell wall of *M. oryzae* through chitin degradation and inhibition of glucan synthesis. Then, it attacks the plasma membrane structure and disrupts the permeability of the plasma membrane, leading to the leakage of internal macromolecules and cytotoxicity [17,22,59]. Furthermore, citral disrupts fungal cell division and restrains the energy supply to exert antifungal effects, as displayed in a comparative proteomic analysis [60]. From the perspective of oxidative stress responses, the present study demonstrates that citral generally enhances antioxidant enzyme activities while concurrently inhibiting the mitochondrial respiratory chain. Even worse, citral reduces MMP through the destruction of the mitochondrial membrane, which might block energy production and cause rapid accumulation of ROS in mitochondria. Afterwards, excessive ROS leads to extreme disequilibrium of redox, such as the synchronously decreased ASA, GSH and Mel, causing a series of oxidative damages in *M. oryzae*. In conclusion, through association analysis of transcriptome and metabolome, our study provides new insights into the molecular mechanisms of antifungal natural products in combating plant disease.

However, the present study focused exclusively on in vitro assays to elucidate citral’s antifungal mechanisms on its oxidative stress under controlled conditions; future studies should validate these promising results in rice plant infected with *M. oryzae*. Given the volatile and unstable nature of citral in the environment, we have also been trying to optimize delivery systems and have made some progress in nanoparticles to effectively enhance citral’s environmental stability [61] Translational application of citral as an advancing eco-friendly pesticide in fields still requires further efforts.

## 4. Materials and Methods

### 4.1. Chemicals, Strain, Culture Media and Reagents

*M. oryzae* ZB08 isolated from rice neck blast was provided by Institute of Crop Protection, Guizhou University. The strain was propagated in potato dextrose agar (PDA) at a constant temperature of 28 °C for optimal growth and preserved at 4 °C. Natural citral (97%, cis and trans) was purchased from Shanghai Macklin Biochemical Technology Co. Ltd. (Shanghai, China) and dissolved in acetone to make a 10 mg/mL stock solution, which was stored at 4 °C for one week of usage. PDA was obtained from Shunyou Shanghai Biotechnology Co. Ltd. (Shanghai, China), while potato dextrose broth (PDB) was made from 200 g fresh potatoes and 20 g dextrose in 1 L distilled water. Both PDA and PDB were sterilized at 121 °C for 15 min to culture the fungi. Assay kits to determine enzymatic activities, compound contents and fluorescence observation were purchased from Beijing Solarbio Science & Technology Co. Ltd. (Beijing, China). All chemicals were analytical-grade.

### 4.2. Effect of Citral on Antioxidant Enzyme Activity

A 5 mm-diameter mycelia plug of *M. oryzae* ZB08 was inoculated to PDA plates and incubated upside down at 28 °C for 7 d. After that, the fungi was cut at the most active growing edge to inoculate in PDB and shaken at 28 °C, 180 rpm for 72 h. The citral stock solution was dispersed as an emulsion in distilled water containing 0.5% (*v*/*v*) Tween 80 and added to PDB to make different concentration while controls received the equal amount of distilled water containing 0.5% Tween. The mycelia were collected by filtration with a four-lay gauze, and 1 g was weighed and exposed to different concentration of citral (0, EC_25_ and EC_75_, equal to 0 µg/mL, 19.83 µg/mL, 110.95 µg/mL) under agitation in an incubator shaker for 0, 1, 2, 4 and 6 h. Then, the culture was centrifuged at 4000× *g* for 15 min, and the mycelia were rinsed with distilled water 3 times for further testing. The effect of citral on antioxidant enzymes of *M. oryzae* was investigated based on the manufacturer’s guidelines.

### 4.3. Determination of Mitochondrial Respiration Complex I, II and III Activity

The *M. oryzae* ZB08 mycelia were incubated with citral and collected as described above. After being rinsed with PBS 3 times, the mycelia were weighed, and 0.1 g mycelia were added to 1.0 mL of extraction solution, followed by quick homogenization on ice using a mortar. After centrifuging at 600 g, 4 °C for 10 min, the supernatant was retained and centrifuged again at 11,000× *g*, 4 °C for 15 min to obtain the precipitate for complex I enzyme activity determination. The subsequent steps were conducted and the activity calculation was performed by following the manufacturer’s instructions of Mt complex I assay kit, Mt complex II assay kit and Mt complex III assay kit (Solarbio Beijing, Beijing, China), respectively.

### 4.4. Determination of ATP Content in Energy Metabolism

The intracellular ATP content was determined by Nicotinamide adenine dinucleotide phoaphate (NADPH), proportional to ATP content, thus reflecting ATP content. The *M. oryzae* mycelia was incubated with citral at various concentrations and prepared as described above. After that, 1 mL of extraction solution was added to the mycelia and homogenized in an ice bath, followed by centrifuge at 8000× *g*, 4 °C for 10 min. The supernatant was transferred to another EP tube, and then 500 µL chloroform was added and vortex thoroughly. After that, the suspension was centrifuged 10,000× *g* at 4 °C for 3 min, and the supernatant was transferred to a new EP tube and placed on ice for measurement according to the guidelines of the ATP content assay kit (Solarbio, Beijing, China).

### 4.5. Imaging of Reactive Oxygen Species (ROS)

The intracellular ROS levels in *M. oryzae* mycelia were determined by a redox-sensitive fluorescent probe DCFH-DA. As described in Section 4.3, 1 g of mycelia cells were incubated with/without citral at 28 ± 2 °C for 4 h, 0.1 g of the mycelia of *M. oryzae* were accurately weighed and mixed with the reagents supplied in the kit to homogenize on ice bath. After centrifuge twice, the mycelia cells in precipitate were mixed with DCFH-DA (final concentration of 10 µM) and incubated for 30 min at 30 °C. After incubation, the cells were washed and resuspended in 0.5 mL of PBS buffer (pH 7.4) and observed with an ECLIPSE Ni-U microscope (Nikon, Tokyo, Japan). Details can be seen in the instruction of ROS assay kit (Solarbio Beijing, China).

### 4.6. Assay of Mitochondrial Membrane Potential (MMP)

The mitochondrial membrane potential detection kit (Solarbio, Beijing, China) is a fluorescent probe that uses JC-1 to rapidly and sensitively detect the influence of citral on Δψm of *M. oryzae*. The mycelia were incubated with citral at concentration of 0, EC_75_ for 4 h. Then, they were collected, washed with PBS and stained with JC-1 for 15 min at room temperature in the dark. After that, the mycelia loaded with probe were rinsed with PBS, and a few mycelia were mounted on a microscope slide to observe and image under a fluorescence microscope ECLIPSE Ni-U (Nikon, Tokyo, Japan) with the excitation and emission wavelength as 529 and 590 nm, respectively. JC-1 monomeric form showed green fluorescence, and JC-1 aggregates showed red fluorescence.

### 4.7. Transcriptome Analysis

The ZB08 strain was activated and incubated in PDB as described above. Mycelia were collected by filtering the culture through four layers of sterile gauze, and then incubated with citral at a concentration of EC_75_ in PDB for 4 h. The control group (0 µg/mL) received the equivalent solvent of citral treatment. After that, the culture was collected in 50 mL EP tubes, and the mycelia were rinsed with 1× PBS three times by centrifugation. Finally, the mycelia were quickly frozen with liquid nitrogen and delivered to Novogene Bioinformatics Technology Co. Ltd. (Beijing, China) for RNA sequencing. The fungal samples were named CK-1, CK-2, CK-3 and Citral-1, Citral-2 and Citral-3 for CK group and citral-treated group, respectively. In the company, total RNAs was extracted using the Trizol reagent, and the RNA integrity and DNA contamination was preliminarily measured by agrose gel electrophoresis. Afterwards, RNA concentration was accurately determined with Qubit2.0 (Life Technologies, Carlsbad, CA, USA) and RNA integrity was accurately measured with Agilent 2100 bioanalyzer (Agilent Technologies, Santa Clara, CA, USA). High quality total mRNA samples were enriched with Oligo (dT) magnetic beads by utilizing the structural feature that eukaryotic mRNA have polyA tails, and mRNAs were subsequently reverse transcribed into cDNA and used in cDNA library construction.

After the library quality control, different libraries were pooled according to the target offline data volume and sequenced using the Illumina HiSeq platform. The offline data was filtered to obtain Clean Data, which was then analyzed by sequence alignment with the specified reference genome to obtain Mapped Data. In this study, clean reads obtained by sequencing were compared with the reference genomic sequence of *M. oryzae* (70-15). Based on the mapped data, structural level analysis such as alternative splicing analysis, new gene discovery, and gene structure optimization were performed; on the other hand, based on the expression levels of genes in different samples or sample groups, differential expression analysis, functional annotation of differentially expressed genes, and functional enrichment analysis, GO and KEGG, were performed as previously described [59]. Based on the gene expression levels of each sample, treatment with citral induced a total of 466 differentially expressed genes (DEGs), which were selected with condition of |log2Fold Change| ≥ 1 and the false discovery rate (FDR) < 0.05.

### 4.8. Widely-Targeted Metabolite Profiling

Mycelia samples of *M. oryzae* were obtained simultaneously by citral treatment as described in transcriptomic analysis and their replicates were named CK-m1, CK-m2, CK-m3 and Citral-m1, Citral-m2 and Citral-m3 for CK group and citral-treated group, respectively. The harvested mycelia were quickly frozen in liquid nitrogen and delivered to Wuhan Metware Biotechnology Co., Ltd. (Wuhan, China) for widely targeted metablome profiling. Briefly, about 50 mg mycelia was homogenized with a zirconia bead for 30 s, 4 times at 30 Hz under frozen condition, then 1 mL of 70% methanol internal standard extraction solution to the homogenized centrifuge tube to extract metabolites overnight. After centrifugation, a final 200 µL supernatant was loaded into liner of the corresponding injection bottle for analysis using an LC-ESI-MS/MS system [62]. Multiple Reaction Monitoring data acquisition was operated in positive and negative ion mode and controlled by Analyst 1.6.3 software (Sciex, Fort Collins, CO, Canada).

Unsupervised PCA (principal component analysis) was performed by statistics function prcomp within R 4.4.3 (www.r-project.org). The data was unit variance scaled before unsupervised PCA. The HCA (hierarchical cluster analysis) results of samples and metabolites were presented as heatmaps with dendrograms, while pearson correlation coefficients (PCC) between samples were calculated by the cor function in R and presented as only heatmaps. Both HCA and PCC were carried out by R 4.4.3 package ComplexHeatmap. For HCA, normalized signal intensities of metabolites (unit variance scaling) were visualized as a color spectrum. Significantly regulated metabolites between groups were determined by VIP ≥ 1 and absolute Log2FC (fold change) ≥ 1. VIP values were extracted from the OPLS-DA result, which also contain score plots and permutation plots, generated using R 4.4.3 package MetaboAnalystR. The data was log-transformed (log2) and mean-centered before OPLS-DA. In order to avoid overfitting, a permutation test (200 permutations) was performed. Identified metabolites were annotated using KEGG Compound database (http://www.kegg.jp/kegg/compound/ (accessed on 13 February 2025)); annotated metabolites were then mapped to the KEGG Pathway database (http://www.kegg.jp/kegg/ pathway. html (accessed on 14 February 2025)). Pathways with significantly regulated metabolites were then fed into MSEA (metabolite set enrichment analysis), and their significance was determined by the hypergeometric test’s *p*-values [63].

### 4.9. Statistical Analysis

All experiments were performed at least in triplicate, and data was expressed as the mean ± SD by measuring three independent biological replicates. All figures were plotted using Sigmaplot (version 14.0). Analysis of variance (ANOVA) and significance analysis were performed using SPSS 26.0 (SPSS Inc., Chicago, IL, USA) followed by *t* test or Dunnett’s test to determine significant differences at *p* < 0.05 level.

## Figures and Tables

**Table 1:** Differential metabolites (DAMs) identified in citral-treated to control mycelia of *M. oryzae*.

Compounds	Index	VIP	Log2FC
**Organic acids**			
2-Methylsuccinic Acid	MEDN0285	1.389470915	1.457618
Glutaric Acid	MEDN0314	1.389470915	1.457618
Phenylpyruvic Acid	MEDN0339	1.391018692	1.420597
Ethylmalonate	MEDN0413	1.389470915	1.457618
2-Furanoic Acid	MEDP0288	1.388682424	−1.13016
L-Dihydroorotic Acid	MEDP0321	1.337825865	−1.11059
Cinnamic Acid	MEDP0332	1.386860535	−1.23325
Folic acid	MEDP1231	1.389345578	−1.35392
**Benzenes**			
2-(Formylamino)Benzoic Acid	MEDN0415	1.384302381	−1.03285
(3,4-Dimethoxyphenyl) Acetic Acid	MEDP0109	1.337700645	−1.72077
2-Methoxybenzoic Acid	MEDP0110	1.391767699	5.030013
Anisic acid	MEDP0647	1.37730052	2.059254
4-Methylbenzoic acid	MEDP0846	1.393335542	12.7864
**Amino acids**			
Glutathione Reduced form	MEDP0044	1.241257863	−1.07454
N-Acetylthreonine	MEDP0386	1.383168672	1.174758
Cys-Gly	MEDP0409	1.226508329	−1.12269
N-Acetylphenylalanine	MEDP0586	1.391699227	1.769527
**Nucleotides**			
Guanosine 3′,5′-Cyclic Monophosphate	MEDN0161	1.349898801	−1.68473
2′-Deoxycytidine-5′-Monophosphate	MEDN0452	1.201755767	−1.07781
7-Methylguanine	MEDP0381	1.356336443	−1.00657
**CoEnzyme and vitamins**			
Vitamin D3	MEDN0241	1.311694478	1.54347
Biotin	MEDP0143	1.313201607	−1.37375
L-Ascorbate	MEDP0239	1.291994893	−1.19252
**FA**			
Dodecanedioic Aicd	MEDP0308	1.392355211	−1.97864
Punicic Acid	MEDP0429	1.221991813	−1.0298
**Hormones**			
Melatonin	MEDN0208	1.392900256	−11.2892
3,3’,5-Triiodo-L-Thyronine	MEDP0184	1.390251632	−1.15749
**Heterocyclic compounds**			
Isoxanthopterin	MEDP0384	1.38384504	−1.03506
Imidazoleacetic acid	MEDP0425	1.391592134	4.480622
**GL**			
Glycine linoleate	MEDN1288	1.365872365	−1.27265

## Data Availability

The transcriptomic raw data has been deposited in the, China National Center for Bioinformation/Beijing Institute of Genomics, Chinese Academy of Sciences (https://ngdc.cncb.ac.cn/gsa/browse/CRA027070 (accessed on 25 June 2025)). The metabolomic raw data has been deposited in the OMIX, China National Center for Bioinformation/Beijing Institute of Genomics, Chinese Academy of Sciences (https://ngdc.cncb.ac.cn/omix/release/OMIX010627 (accessed on 18 June 2025)).

## Supplementary Materials

The following supporting information can be downloaded at: https://www.mdpi.com/article/10.3390/plants14132001/s1, Table S1. Sequencing data assessment statistics of *M. oryzae* in response to citral. Figure S1. Volcano-plot of DEGs in citral treated mycelia and control group of *M. oryzae*. Figure S2. Heatmap of hierarchical clustering of DEGs in citral treated mycelia and control mycelia of *M. oryzae*. Table S2. Primer sequences of selected genes for qRT-PCR validation of RNA-seq. Figure S3. Relative expression level validation of DEGs by quantitative real-time polymerase chain reaction (qRT-PCR). Figure S4. GO functional enrichment of DEGs citral treated mycelia and control group of *M. oryzae*. Figure S5. PCA and OPLS-DA analysis of Citral/CK metabolites of *M. oryzae*. Figure S6. Heatmap of hierarchical clustering of DAMs in *M. oryzae*. Figure S7. KEGG enrichment *p* value histogram of differential genes and metabolites in citral treated group and control group of *M. oryzae*.

## References

[1] Deng Y., Zhai K., Zhen X., Yang D., Zhu X., Liu J., He Z. (2017). Epigenetic regulation of antagonistic receptors confers rice blast resistance with yield balance. Science.

[2] Talbot N.J. (2003). On the trail of a cereal killer: Investigating the biology of *Magnaporthe grisea*. Annu. Rev. Microbiol..

[3] Kumar S., Kashyap P.L., Mahapatra S., Jasrotia P., Singh G.P. (2021). New and emerging technologies for detecting *Magnaporthe oryzae* causing blast disease in crop plants. Crop Prot..

[4] Galhano R., Talbot N.J. (2011). The biology of blast: Understanding how *Magnaporthe oryzae* invades rice plants. Fungal Biol. Rev..

[5] Mohiddin F.A., Bhat N.A., Wani S.H., Bhat A.H., Ahanger M.A., Shikari A.B., Sofi N.R., Parveen S., Khan G.H., Bashir Z. (2021). Combination of strobilurin and triazole chemicals for the management of blast disease in mushk budji-aromatic rice. J. Fungi.

[6] Ghazanfar M.U., Wakil W., Sahi S.T., Kaku K.S. (2009). Influence of various fungicides on the management of rice blast disease. Mycopath..

[7] Chen Y., Zhang Y., Yao J., Li Y., Yang X., Wang W., Zhang A., Gao T. (2013). Frequency distribution of sensitivity of *ustilaginoidea virens* to four ebi fungicides, prochloraz, difenoconazole, propiconazole and tebuconazole, and their efficacy in controlling rice false smut in anhui province of china. Phytoparasitica.

[8] Xin W., Mao Y., Lu F., Li T., Wang J., Duan Y.B., Zhou M. (2020). In vitro fungicidal activity and in planta control efficacy of coumoxy -strobin against *Magnaporthe oryzae*. Pestic. Biochem. Physiol..

[9] Adukwu E.C., Bowles M., Edwards-Jones V., Bone H. (2016). Antimicrobial activity, cytotoxicity and chemical analysis of lemongrass essential oil (*Cymbopogon flexuosus*) and pure citral. Appl. Microbiol. Biotechnol..

[10] Saeed K., Pasha I., Jahangir Chughtai M.F., Ali Z., Bukhari H., Zuhair M. (2022). Application of essential oils in food industry: Challenges and innovation. J. Essent. Oil Res..

[11] Kim I.H., Oh Y.A., Lee H., Song K.B., Min S.C. (2014). Grape berry coatings of lemongrass oil-incorporating nanoemulsion. Lwt Food Sci. Technol..

[12] Wei L., Chen C., Wan C., Chen M., Chen J. (2021). Citral delays postharvest senescence of kiwifruit by enhancing antioxidant capacity under cold storage. J. Food Qual..

[13] Gao X., Hu X., Mo F., Ding Y., Li M., Li R. (2022). Repellency mechanism of natural guar gum-based film incorporated with citral against brown planthopper, *Nilaparvata lugens* (Stål) (Hemiptera: Delphacidae). Int. J. Mol. Sci..

[14] Singh S., Kurmi A., Singh V., Singh M.K., Mishra S., Shankar U., Savita A., Gupta H., Yadav N.P., Sakia D. (2024). *Cymbopogon distans*: A source of essential oil with potential antibacterial, antifungal, and mosquito-repelling properties. Food Biosci..

[15] Eraslan E.C., Çırçırlı B., Özkan A., Akgül H. (2021). Anticancer mechanisms of action of macrofungus extracts. Eurasian J. Med. Biol. Sci..

[16] Mohammed F.S., Sevindik E., Uysal I., Sevindik M. (2024). Miracle plant *Moringa oleifera* Lam.: Nutritional, mineral, essential oil contents and biological activities. Vegetos.

[17] Li R., Wu X., Yin X., Liang J., Li M. (2014). The natural product citral can cause significant damage to the hyphal cell walls of *Magnaporthe grisea*. Molecules.

[18] Kang S., Li X., Xing Z., Liu X., Bai X., Yang Y., Guo D., Xia X., Zhang C., Shi C. (2022). Antibacterial effect of citral on *yersinia enterocolitica* and its mechanism. Food Control.

[19] Dai J., Bai M., Li C., San Cheang W., Cui H., Lin L. (2023). Antibacterial properties of citral against *Staphylococcus aureus*: From membrane damage to metabolic inhibition. Food Biosci..

[20] Richter S., Gatto B., Fabris D., Takao K.I., Kobayashi S., Palumbo M. (2003). Clerocidin alkylates DNA through its epoxide function: Evidence for a fine tuned mechanism of action. Nucleic Acids Res..

[21] Lima I.O., de Medeiros Nóbrega F., de Oliveira W.A., de Oliveira Lima E., Albuquerque Menezes E., Afrânio Cunha F., de Fátima Formiga Melo Diniz M. (2012). Anti-*Candida albicans* effectiveness of citral and investigation of mode of action. Pharm. Biol..

[22] Li R., Wu X., Yin X., Long Y., Li M. (2015). Naturally produced citral can significantly inhibit normal physiology and induce cytotoxicity on *Magnaporthe grisea*. Pestic. Biochem. Phys..

[23] Ju J., Xie Y., Yu H., Guo Y., Cheng Y., Zhang R., Yao W. (2020). Synergistic inhibition effect of citral and eugenol against *Aspergillus niger* and their application in bread preservation. Food Chem..

[24] Zhang Y., Wei J., Chen H., Song Z., Guo H., Yuan Y., Yue T. (2020). Antibacterial activity of essential oils against *Stenotrophomonas maltophilia* and the effect of citral on cell membrane. Lwt.

[25] Wang Y., Zhang Z., Jing C., Mou G., Zhang W., Jin Y., Qin L., An J., Zhang S., Liu Y. (2024). Antifungal Effects and Postharvest Diseases Control Potential of E, E-2, 4-Nonadienal against *Rhizopus stolonifer*. J. Agric. Food Chem..

[26] Zheng S., Jing G., Wang X., Ouyang Q., Jia L., Tao N. (2015). Citral exerts its antifungal activity against *Penicillium digitatum* by affecting the mitochondrial morphology and function. Food Chem..

[27] OuYang Q., Tao N., Zhang M. (2018). A damaged oxidative phosphorylation mechanism is involved in the antifungal activity of citral against *Penicillium digitatum*. Front. Microbiol..

[28] Wang Y., Feng K., Yang H., Zhang Z., Yuan Y., Yue T. (2018). Effect of cinnamaldehyde and citral combination on transcriptional profile, growth, oxidative damage and patulin biosynthesis of *Penicillium expansum*. Front. Microbiol..

[29] Wang L., Jiang N., Wang D., Wang M. (2019). Effects of essential oil citral on the growth, mycotoxin biosynthesis and transcriptomic profile of *Alternaria alternata*. Toxins.

[30] Wani M.Y., Ahmad A., Aqlan F.M., Al-Bogami A.S. (2021). Citral derivative activates cell cycle arrest and apoptosis signaling pathways in *Candida albicans* by generating oxidative stress. Bioorg. Chem..

[31] Semenza G.L. (2007). Oxygen-dependent regulation of mitochondrial respiration by hypoxia-inducible factor 1. Biochem. J..

[32] Pandyra A., Mullen P.J., Kalkat M., Yu R., Pong J.T., Li Z., Trudel S., Lang K.S., Minden M.D., Schimmer A.D. (2014). Immediate Utility of Two Approved Agents to Target Both the Metabolic Mevalonate Pathway and Its Restorative Feedback Loop. Cancer Res..

[33] Dawson D.A., Pöch G. (2017). Evaluation of consistency for multiple experiments of a single combination in the time- dependence mixture toxicity assay. Toxicol. Mech. Methods.

[34] Luiz R.C., Cecchini A.L. (2021). Mitochondria as a Target for Monoterpenes. Mitochondrial Physiology and Vegetal Molecules.

[35] Hasanuzzaman M., Bhuyan M.B., Zulfiqar F., Raza A., Mohsin S.M., Mahmud J.A., Fujita M., Fotopoulos V. (2020). Reactive oxygen species and antioxidant defense in plants under abiotic stress: Revisiting the crucial role of a universal defense regulator. Antioxidants.

[36] Hwang J.H., Hwang I.S., Liu Q.H., Woo E.R., Lee D.G. (2012). (+)-Medioresinol leads to intracellular ROS accumulation and mitochondria-mediated apoptotic cell death in *Candida albicans*. Biochimie.

[37] Mittler R. (2002). Oxidative stress, antioxidants and stress tolerance. Trends Plant Sci..

[38] Lushchak V.I. (2011). Adaptive response to oxidative stress: Bacteria, fungi, plants and animals. Comp. Biochem. Phys. C.

[39] de Arruda Grossklaus D., Bailão A.M., Rezende T.C.V., Borges C.L., de Oliveira M.A.P., Parente J.A., de Almeida Soares C.M. (2013). Response to oxidative stress in *Paracoccidioides* yeast cells as determined by proteomic analysis. Microbes Infect..

[40] Mo F., Hu X., Ding Y., Li R., Long Y., Wu X., Li M. (2021). Naturally produced magnolol can significantly damage the plasma membrane of *Rhizoctonia solani*. Pestic. Biochem. Phys..

[41] Harding H.P., Zhang Y., Zeng H., Novoa I., Lu P.D., Calfon M., Sadri N., Yun C., Popko B., Paules R. (2003). An integrated stress response regulates amino acid metabolism and resistance to oxidative stress. Mol. Cell.

[42] Daddam J.R., Sura M., Vocelle D., Laguna J.G., Gallagher K., Zhou Z. (2025). The supply of branched-chain amino acids and branched-chain keto acids alter lipid metabolism, oxidative stress, and apoptosis in primary bovine hepatocytes. J. Nutr. Biochem..

[43] Yang W., Liu D., Gao P., Wu Q., Li Z., Li S., Zhu L. (2024). Oxidative stress and metabolic process responses of *Chlorella pyrenoidosa* to nanoplastic exposure: Insights from integrated analysis of transcriptomics and metabolomics. Environ. Pollut..

[44] Chen P., An B., Hu Y., Tao Y. (2025). 2,4-Bisphenol S triggers physiological changes, oxidative stress and lipidome alterations in Gram-positive *Enterococcus faecalis* at environmental concentrations. Environ. Pollut..

[45] Ramzan T., Shahbaz M., Maqsood M.F., Zulfiqar U., Saman R.U., Lili N., Irshad M., Maqsood S., Haider A., Shahzad B. (2023). Phenylalanine supply alleviates the drought stress in mustard (*Brassica campestris*) by modulating plant growth, photosynthesis, and antioxidant defense system. Plant Physiol. Biochem..

[46] Dong Q., Li D., Wu Y., Zhou C., Lin Y., Miao P., Li J., Pan C. (2023). Exogenous nanoselenium alleviates imidacloprid-induced oxidative stress toxicity by improving phenylpropanoid metabolism and antioxidant defense system in *Perilla frutescens* (L.) Britt. J. Plant Physiol..

[47] Mishra V., Tripathi D.K., Rai P., Sharma S., Singh V.P. (2024). Regulation of arsenate stress by nitric oxide and hydrogen sulfide in *Oryza sativa* seedlings: Implication of sulfur assimilation, glutathione biosynthesis, and the ascorbate-glutathione cycle and its genes. Plant Physiol. Biochem..

[48] Husain T., Prasad S.M., Singh V.P. (2024). Ethylene and hydrogen sulfide regulate hexavalent chromium toxicity in two pulse crops: Implication on growth, photosynthetic activity, oxidative stress and ascorbate glutathione cycle. Plant Physiol. Biochem..

[49] Chen Z., Liu J., Gao B., Sillanpää M. (2024). Sterilization mechanism of CuCeOx on fungus: Oxidative damage and energy metabolism disequilibrium. J. Environ. Chem. Eng..

[50] Asadi E., Maresca V., Sorbo S., Keramat B., Basile A. (2017). Effects of triacontanol on ascorbate-glutathione cycle in *Brassica napus* L. exposed to cadmium-induced oxidative stress. Ecotox. Environ. Safe.

[51] Singh V.P., Srivastava P.K., Prasad S.M. (2012). Differential effect of UV-B radiation on growth, oxidative stress and ascorbate glutathione cycle in two cyanobacteria under copper toxicity. Plant Physiol. Biochem..

[52] Khan M.N. (2023). Melatonin regulates mitochondrial enzymes and ascorbate-glutathione system during plant responses to drought stress through involving endogenous calcium. S. Afr. J. Bot..

[53] Wang G., Chen X., Zhang C., Li M., Sun C., Zhan N., Huang X., Li T., Deng W. (2021). Biosynthetic pathway and the potential role of melatonin at different abiotic stressors and developmental stages in *Tolypocladium guangdongense*. Front. Microbiol..

[54] Morcillo-Parra M.Á., Beltran G., Mas A., Torija M.J. (2020). Effect of several nutrients and environmental conditions on intracellular melatonin synthesis in *Saccharomyces cerevisiae*. Microorganisms.

[55] Asdullah H.U., Chen F., Hassan M.A., Abbas A., Sajad S., Rafiq M., Chen Y. (2024). Recent advances and role of melatonin in post-harvest quality preservation of shiitake (*Lentinula edodes*). Front. Nutr..

[56] Gao Y., Wang Y., Qian J., Si W., Tan Q., Xu J., Zhao Y. (2020). Melatonin enhances the cadmium tolerance of mushrooms through antioxidant-related metabolites and enzymes. Food Chem..

[57] Fanjul-Moles M.L., López-Riquelme G.O. (2016). Relationship between oxidative stress, circadian rhythms, and AMD. Oxid. Med. Cell Longev..

[58] Jiménez A., Sevilla F., Martí M.C. (2021). Reactive oxygen species homeostasis and circadian rhythms in plants. J. Exp. Bot.

[59] Song X., Zhao Q., Zhou A., Wen X., Li M., Li R., Liao X., Xu T. (2021). The antifungal effects of citral on Magnaporthe oryzae occur via modulation of chitin content as revealed by RNA-Seq analysis. J. Fungi.

[60] Zhao Q., Ding Y., Song X., Liu S., Li M., Li R., Ruan H. (2021). Proteomic analysis reveals that naturally produced citral can significantly disturb physiological and metabolic processes in the rice blast fungus *Magnaporthe oryzae*. Pestic. Biochem. Phys..

[61] Ding Y., Yuan J., Wu S., Hu K., Ma Y., Gao Y., Li M., Li R. (2024). pH/chitinase dual stimuli-responsive essential oil-delivery system based on mesoporous silica nanoparticles for control of rice blast. Pest. Manag. Sci..

[62] Chen W., Gong L., Guo Z., Wang W., Zhang H., Liu X., Yu S., Xiong L., Luo J. (2013). A novel integrated method for large-scale detection, identification, and quantification of widely targeted metabolites: Application in the study of rice metabolomics. Mol. Plant.

[63] Yan Y., Tang J., Yuan Q., Liu H., Huang J., Hsiang T., Bao C., Zheng L. (2022). Ornithine decarboxylase of the fungal pathogen *Colletotrichum higginsianum* plays an important role in regulating global metabolic pathways and virulence. Environ. Microbiol..

