# Supplementary material for: Investigating the Mechanisms Underlying Citral-Induced Oxidative Stress and Its Contribution to Antifungal Efficacy on Magnaporthe oryzae Through a Multi-Omics Approach
Source: Plants (Basel). 2025 Jun 30;14(13):2001. doi: 10.3390/plants14132001 (PMC12252439; doi:10.3390/plants14132001)
Supplement: Supplementary file 1 [file plants-14-02001-s001.zip › plants-3695398-supplementary.pdf]

Table s1. Sequencing data assessment statistics of *M. oryzae* in response to citral.

Sample	Raw Reads	Clean Reads	Clean Base (G)	Clean Reads Q20 (%)	Clean Reads Q30 (%)	Reads mapped	Unique mapped
Citral1	45406278	43751818	6.56	98.33	95.13	42023690(96.05%)	41736810(95.39%)
Citral2	49493408	48168626	7.23	98.34	95.25	46230941(95.98%)	45900982(95.29%)
Citral3	45772006	44365688	6.65	98.36	95.22	42591770(96.00%)	42294144(95.33%)
CK1	44373566	42647334	6.40	98.41	95.33	40911552(95.93%)	40614068(95.23%)
CK2	48878654	47008122	7.05	98.39	95.4	45055462(95.85%)	44723525(95.14%)
CK3	45383492	43916844	6.59	98.38	95.26	42185313(96.06%)	41868507(95.34%)

Q30, the proportion of bases with a quality value greater than 30 to the total number of bases. Unique mapped, reads only mapped to a specific position on the reference genome.

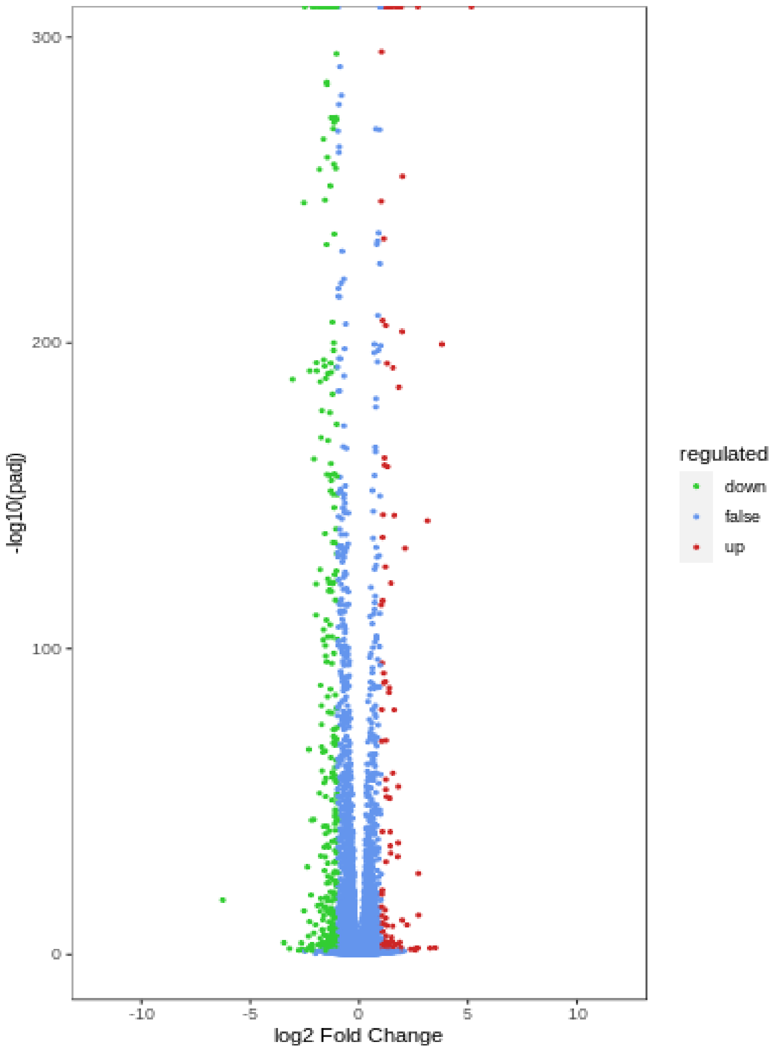

Figure s1. Volcano-plot of DEGs in citral treated mycelia and control group of *M. oryzae*. The abscissa indicates the change of gene expression factor and the ordinate indicates the significance level of differential genes. The red and green markers represent up-regulated genes and down-regulated genes, respectively

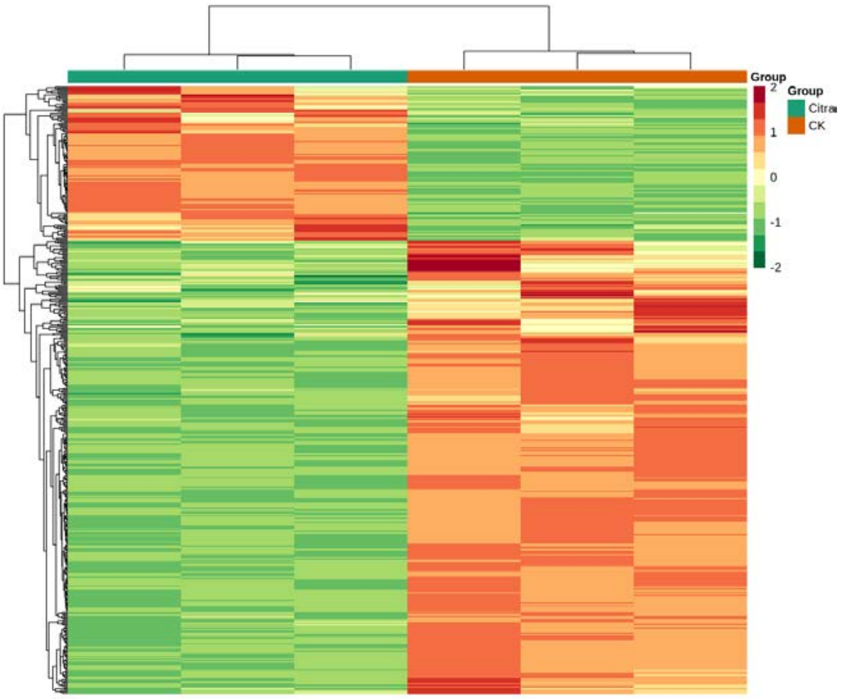

Figure s2. Heatmap of hierarchical clustering of DEGs in citral treated mycelia and control mycelia of *M. oryzae*. The abscissa represents the sample name and the hierarchical clustering result, and the ordinate represents the differential gene and the hierarchical clustering result. Red indicates high expression and green indicates low expression.

Table s2. Primer sequences of selected genes for qRT-PCR validation of RNA-seq.

**Gene Name**	**Forword Primer (5'-3')**	**Reverse primer (5′-3′)**	**Length(bp)**
MGG_12421	GGGATTCGTGGGCTGTGAGG	CGAGACAAACACGGGCTCCTT	124
MGG_13253	GATGGAGCATAATTTCCGATTC	GTGCCTTGGGTGAGTTTGG	95
MGG_07890	CGTTCCGCTACCCGACCTA	CGCCCAGACAAACGCTCTAC	149
MGG_02125	CGGCGACAAGACAATCCA	ACGGCGTCCATGAAGAGC	114
MGG_05984	AACTTCTCAGCACCTTCTTCC	ACCAGACGGGTTCCAAAC	177
MGG_11608	GTCAGGAATGCGAGAACGG	TGATGGTCTGGCAGTAGCG	174
MGG_07800	TGCAACGAGCAACAACAAATC	TCCAAACTGCCCTCACCC	95
MGG_05827	AGCCTGGGCGTACCTAGAGC	TGCGTGGGCATGTCCTTG	141
MGG_01084 (GAPDH)	CGAGTACACCGAGGACGAT	GCTGACGAGCTTGACGAAC	115

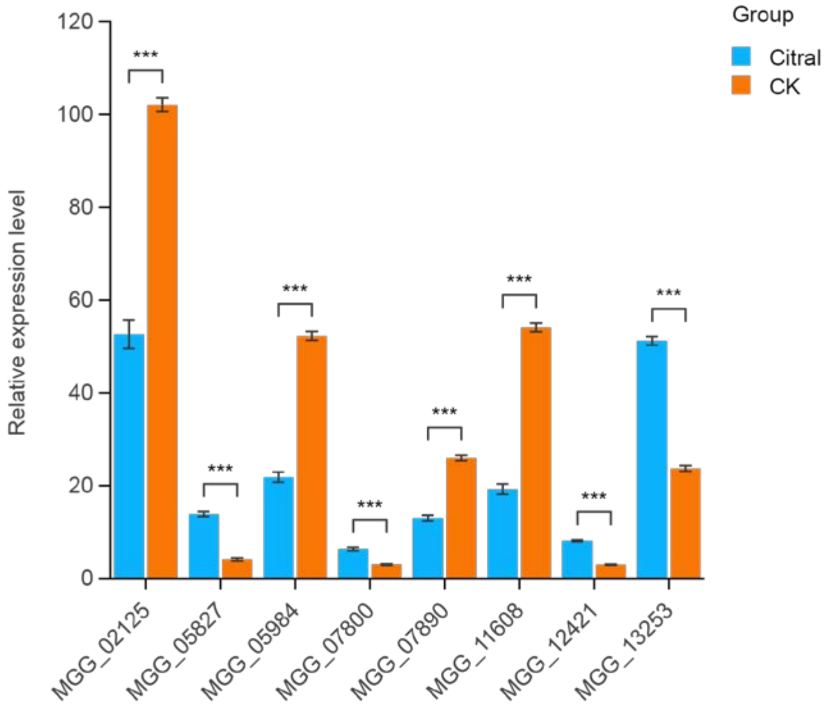

Figure s3. Relative expression level validation of DEGs by quantitative real-time polymerase chain reaction (qRT-PCR). The relative expression levels of mRNA were normalized with internal reference gene (GAPDH) of *M.oryzae*. MGG_02125, MGG_05827, MGG_05984, MGG_07800, MGG_07890, MGG_11608, MGG_12421 and MGG_13253 codes for branched chain amino acid transferase, glutamyl-tRNA (Gln) amidotransferase subunit A, gluconolactonase lactonase, oxoprolinase, aldehyde dehydrogenase, laccase, dimethylglycine and choline dehydrogenase.

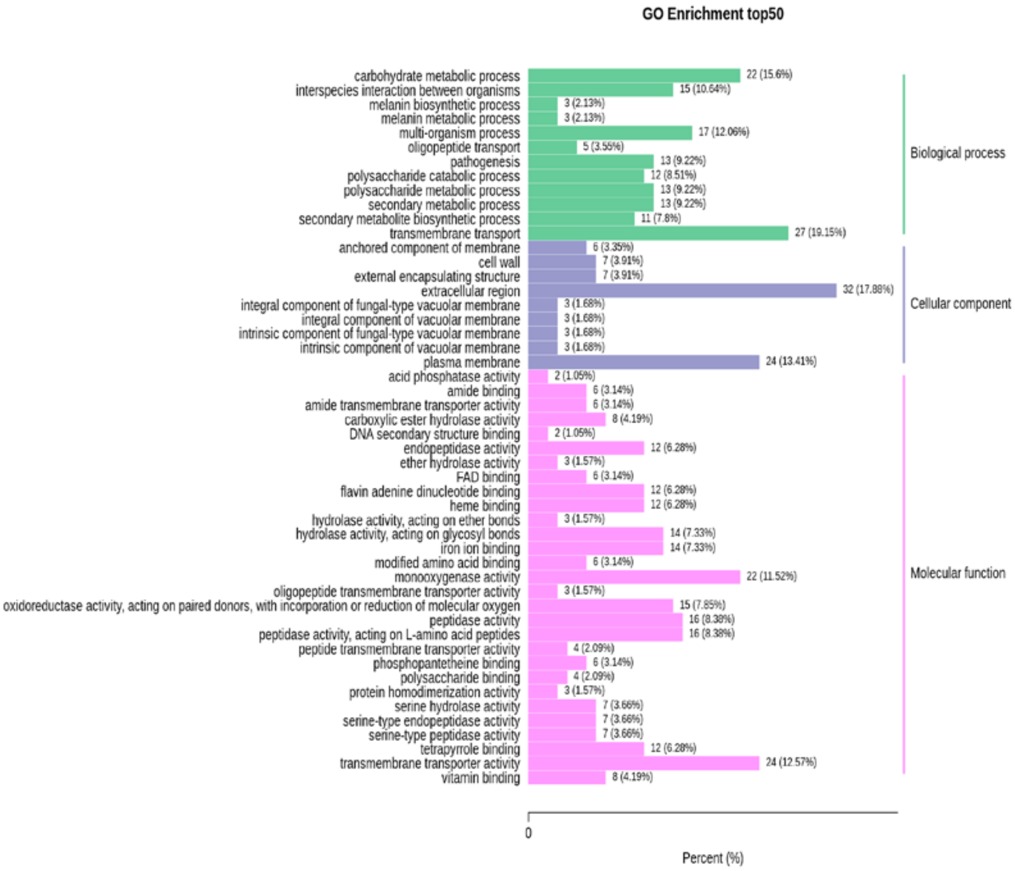

Figure s4. GO functional enrichment of DEGs citral treated mycelia and control group of *M. oryzae*.

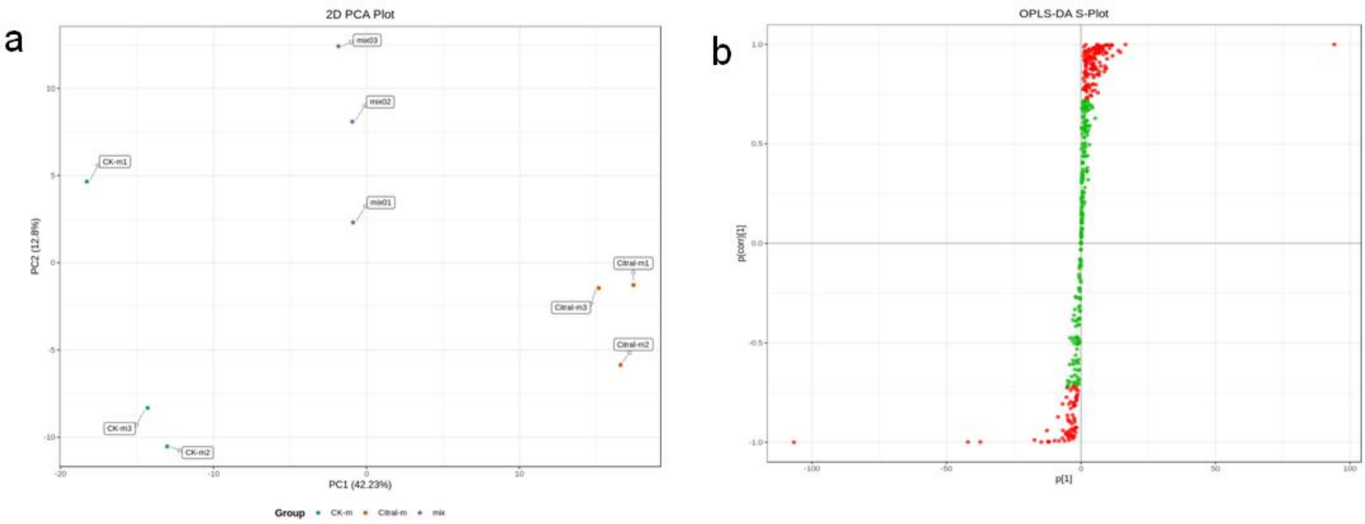

Figure s5. PCA and OPLS-DA analysis of Citral/CK metabolites of *M. oryzae*.

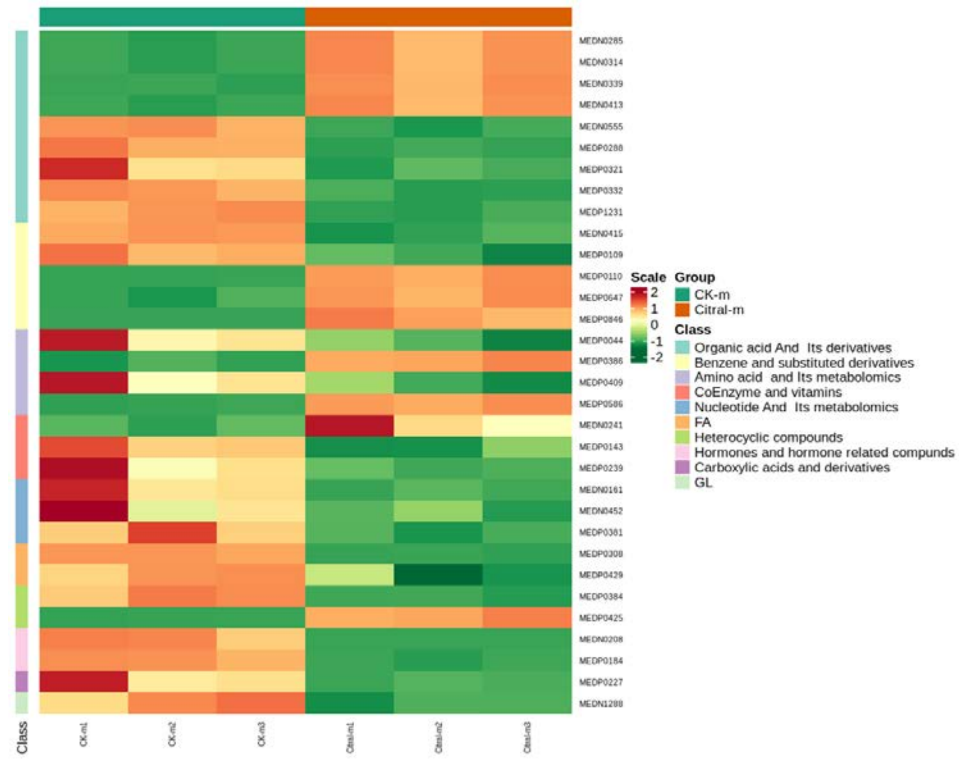

Figure s6. Heatmap of hierarchical clustering of DAMs in *M. oryzae*. The left ends represent clusters of differential metabolites and each rectangular block in the heatmap represents one metabolite.

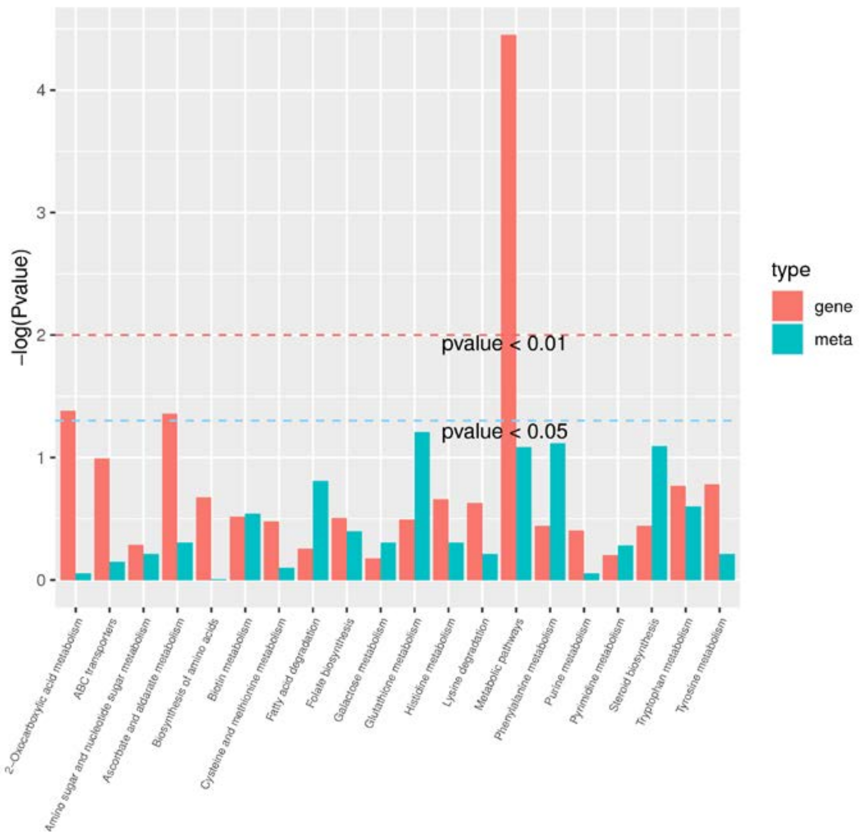

Figure s7. KEGG enrichment p value histogram of differential genes and metabolites in citral treated group and control group of *M. oryzae*.
